# The Role of Metabolites in the Link between DNA Replication and Central Carbon Metabolism in *Escherichia coli*

**DOI:** 10.3390/genes11040447

**Published:** 2020-04-19

**Authors:** Klaudyna Krause, Monika Maciąg-Dorszyńska, Anna Wosinski, Lidia Gaffke, Joanna Morcinek-Orłowska, Estera Rintz, Patrycja Bielańska, Agnieszka Szalewska-Pałasz, Georgi Muskhelishvili, Grzegorz Węgrzyn

**Affiliations:** 1 Department of Bacterial Molecular Genetics, University of Gdansk, 80-308 Gdansk, Poland; klaudyna.krause@phdstud.ug.edu.pl (K.K.); joanna.morcinek-orlowska@phdstud.ug.edu.pl (J.M.-O.); agnieszka.szalewska-palasz@biol.ug.edu.pl (A.S.-P.); 2 Institute of Biochemistry and Biophysics, Polish Academy of Sciences, 80-822 Gdansk, Poland; monika.maciag-dorszynska@biol.ug.edu.pl; 3 Department of Molecular Biology, University of Gdansk, 80-308 Gdansk, Poland; anna.wosinski@gmail.com (A.W.); lidia.gaffke@phdstud.ug.edu.pl (L.G.); estera.rintz@gmail.com (E.R.); patrycjabielanska.98@wp.pl (P.B.); 4 School of Natural Sciences, Agricultural University of Georgia, 0131 Tbilisi, Georgia; g.muskhelishvili@agruni.edu.ge

**Keywords:** DNA replication, central carbon metabolism, metabolites, replication control, suppression of replication mutants

## Abstract

A direct link between DNA replication regulation and central carbon metabolism (CCM) has been previously demonstrated in *Bacillus subtilis* and *Escherichia coli*, as effects of certain mutations in genes coding for replication proteins could be specifically suppressed by particular mutations in genes encoding CCM enzymes. However, specific molecular mechanism(s) of this link remained unknown. In this report, we demonstrate that various CCM metabolites can suppress the effects of mutations in different replication genes of *E. coli* on bacterial growth, cell morphology, and nucleoid localization. This provides evidence that the CCM-replication link is mediated by metabolites rather than direct protein-protein interactions. On the other hand, action of metabolites on DNA replication appears indirect rather than based on direct influence on the replication machinery, as rate of DNA synthesis could not be corrected by metabolites in short-term experiments. This corroborates the recent discovery that in *B. subtilis*, there are multiple links connecting CCM to DNA replication initiation and elongation. Therefore, one may suggest that although different in detail, the molecular mechanisms of CCM-dependent regulation of DNA replication are similar in *E. coli* and *B. subtilis*, making this regulation an important and common constituent of the control of cell physiology in bacteria.

## 1. Introduction

The replication of genetic material is one of the fundamental metabolic processes in the living cell. The genetic stability as well as precise functioning of the organism is dependent on the proper regulation of this process. The copying of the DNA during replication, which precedes cell division, is performed by a complex of various enzymes. These proteins can be divided into different groups: proteins altering the DNA structure, enzymes synthesizing nucleic acids, proteins responsible for the accuracy of copying process, and correction of occurring errors. The complexity of the replication and its dependency of the available energy sources require precise regulation. DNA replication is regulated at many sequential steps, beginning with initiation, through elongation, and to its end—the termination [1].

During the last decades, studies of the replication process helped to characterize the replication factory elements and to understand the role of its components. Nevertheless, until now, the entire picture of the molecular mechanism regulating this fundamental process has not been fully understood. In order to function correctly, the cell needs nutrients providing both the building blocks and energy, and consequently, a tight cooperation between all cellular processes. However, the core of this relationship remains unclear. The key element of this cooperation is central carbon metabolism (CCM). Similar to DNA replication, CCM is indispensable in every organism [1,2]. The reactions of CCM allow the maintenance of cellular homeostasis by keeping the optimal balance between energy production and usage in the cell under various growth conditions. 

Until recently, it was assumed that DNA replication and CCM are connected only indirectly through CCM-depended synthesis of the nucleic acids’ precursors and provision of metabolic energy. However, it has been demonstrated that effects of certain mutations in genes coding for replication proteins could specifically be suppressed by particular mutations in genes encoding CCM enzymes in *Bacillus subtilis* [3]. Since indirect suppression due to variations in bacterial growth rate was excluded, it appeared that there is a direct link between CCM and DNA replication [3]. Indeed, recent studies indicated that there are multiple links connecting CCM to DNA replication initiation and elongation in *B. subtilis* [4]. Other investigations indicated that similar relationships also occur in *Escherichia coli* [5], although differences in detailed effects of CCM on DNA replication between these two bacteria were evident (summarized in [6]). Interestingly, there are also reports suggesting that enzymes of CCM might contribute to regulation of DNA replication in eukaryotic cells, including human cells (for recent reports, see [7,8,9]; for reviews and discussions, see [6,10,11]).

Despite the evidence that DNA replication regulation is directly linked to CCM, the molecular mechanism of this relationship remains unknown. Previous studies indicated that effects of various mutants of *E. coli* could be alleviated by dysfunction of specific genes. The suppression pattern was as follows: the effects of *dnaA46*(ts) mutation could be suppressed by dysfunction of *pta* or *ackA*, effects of *dnaB*(ts) by dysfunction of *pgi* or *pta*, effects of *dnaE486*(ts) by dysfunction of *tktB*, effects of *dnaG*(ts) by dysfunction of *gpmA*, *pta,* or *ackA*, and effects of *dnaN159*(ts) by dysfunction of *pta* or *ackA* [5]. The observed suppression suggested that some metabolic enzymes could modulate replisome properties in response to the physiological state of the cell, moreover, the deficiency of CCM enzymes may lead to accumulation of certain metabolites (replication and CCM genes and enzymes, together with their functions, are listed in Table 1). These metabolites, in the wild-type cells, could be substrates for particular metabolic enzymes, while in mutants lacking specific enzymes, accumulation of the metabolites would result in metabolic disturbance and possible inhibition of growth. The metabolites could also serve as small molecule signals transmitting information about the metabolic condition and causing the necessary adjustments in DNA replication and cell division. To test this hypothesis, we aimed to investigate the effects of carbon pathways metabolites (acetate, pyruvate, succinate, fumarate, malate, lactate, and α-ketoglutarate) on bacterial growth and DNA replication. Metabolites from glycolysis/gluconeogenesis, overflow pathway, and tricarboxylic acid (TCA) cycle were chosen to mimic effects of mutations in particular genes, which were previously reported to suppress the defects observed in replication mutants. 

## 2. Materials and Methods 

### 2.1. Bacterial Strains

*E. coli* MG1655 strain [12] was used in this work as a wild-type control, and other isogenic *dna*(ts) mutants are listed in Table 2. 

### 2.2. Metabolites

The tested compounds were purchased from Sigma Aldrich (Saint Louis, MO, USA). Stock solutions were prepared in distillated water and used to achieve indicated concentrations.

### 2.3. Effects of Metabolites on the Growth of Bacterial Strains

Bacteria were grown in liquid lysogeny broth (LB) medium at 30 °C in flasks. Following dilution (1:100) of overnight cultures with fresh LB, the cells were grown under the same conditions until A_600_ = 0.2. Next, 100 ml of each culture or its dilution were plated on solid LB medium (LB supplemented with 1.5% agar) containing indicated concentration of tested metabolite. The plates were then incubated at either 30 °C (controls) or following semi-restrictive temperatures (determined according to [5]): 39 °C for *dnaA46*(ts), 41 °C for *dnaB8*(ts), 35 °C for *dnaC*(ts), 36.5 °C for *dnaE486*(ts), 34 °C for *dnaG*(ts), and 37.5 °C for *dnaN159*(ts) for 16 h. Colony forming units (CFU) were calculated from plates where the colony number was between 100 and 1000.

### 2.4. Microscopic Analysis 

The optical microscopy analysis was performed as described previously [3]. Briefly, bacteria were grown exponentially at 30 °C until A_600_ = 0.2. Then, the cultures were shifted to semi-restrictive temperatures (as listed in the preceding subsection) for the time equal to five generations (as estimated at 30 °C). After the incubation, cells were stained for 10 min with DAPI (4’,6’-diamidino-2-phenylindole) (Sigma Aldrich, Saint Louis, MO, USA) and SynaptoRed™ (N-(3-triethylammoniopropyl)-4-(6-(4-(diethylamino)phenyl) hexatrienyl) pyridinium dibromide, pyridinium, 4-[6-[4-(diethylamino) phenyl]- 1,3,5-hexatrienyl]-1-[3-(triethylammonio) propyl]-, dibromide) ( Sigma Aldrich, Saint Louis, MO, USA), as described previously [17]. Bacteria were observed under the Leica DMI4000B microscope at the magnification of 100 X and photographed with Leica DFC365FX. The length of 100 randomly chosen cells was measured by ImageJ Ink program and analyzed with GraphPad Prism 5.

### 2.5. Kinetics of DNA Replication In Vivo

Overnight bacterial cultures grown at 30 °C in LB were diluted (1:100), and the incubation was continued until A_600_ = 0.1. Then, [^3^H]thymidine was added to indicated concentration and to radioactivity of 5 μCi/mL, and the culture was transferred to semi-restrictive temperature. At indicated times, samples were placed onto Whatman 3MM paper filters and then immediately transferred to 10% trichloroacetic acid (TCA) for 10 min. Following this incubation, the filters were washed in 5% TCA and 96% ethanol. The filters were dried and their radioactivity was measured in the scintillation counter (Microbeta2^®^, Perkin Elmer, Waltham, MA, USA). 

## 3. Results

### 3.1. Growth Defect Suppression of Replication Mutant Strains by Certain CCM Metabolites

**Figure 1:**
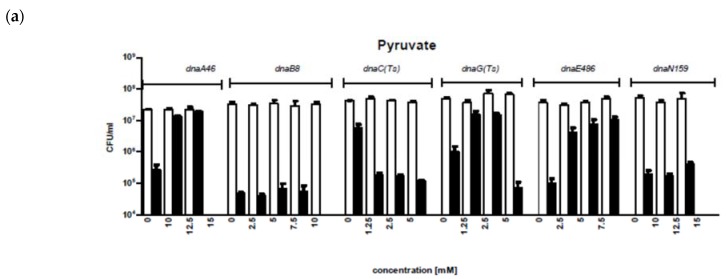

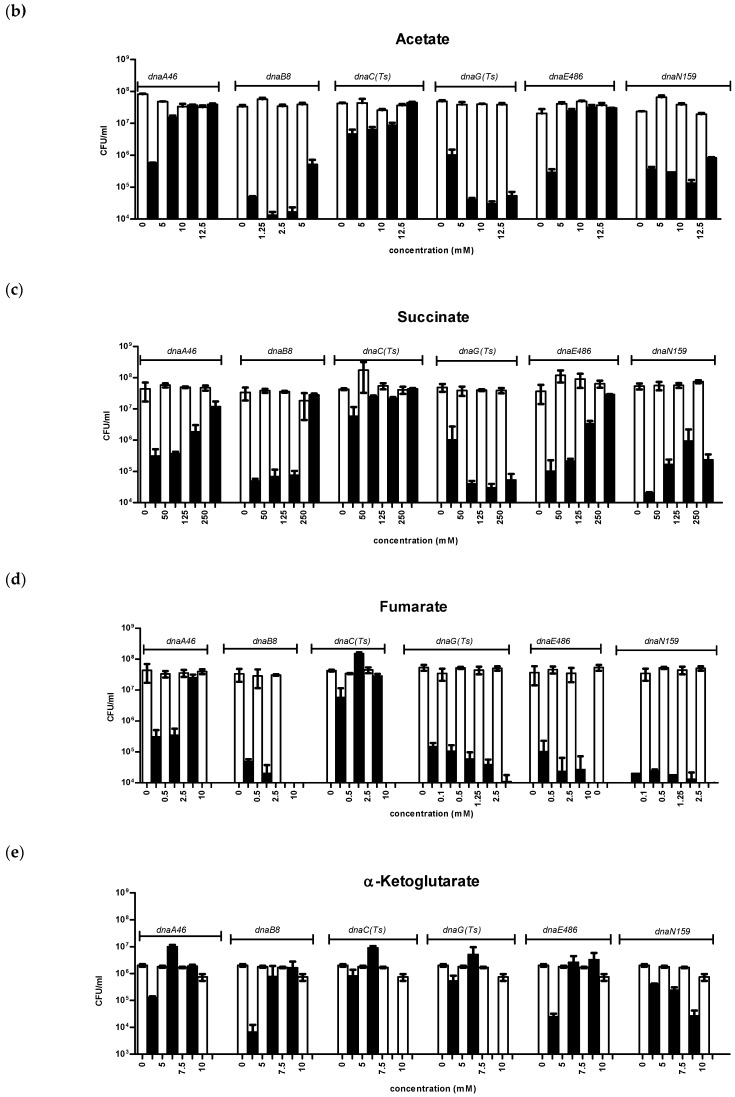

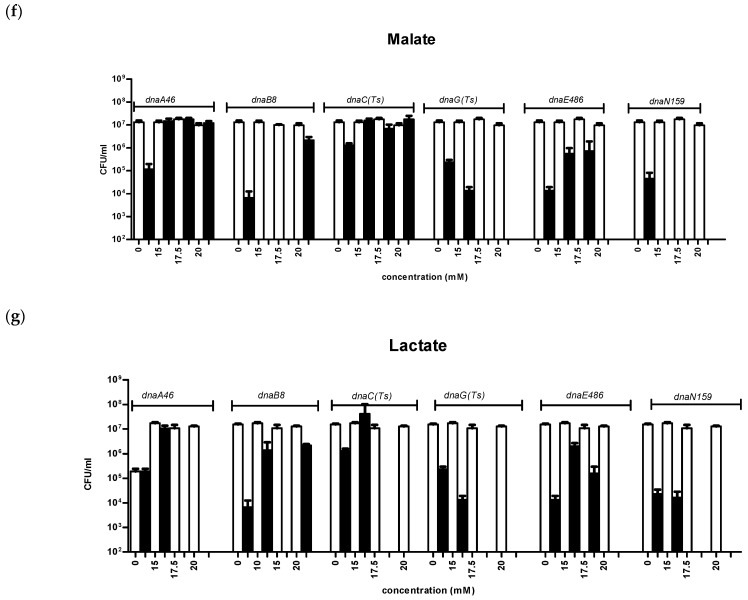
The growth of replication-impaired temperature sensitive mutants in the presence of following CCM metabolites: (**a**) pyruvate, (**b**) acetate, (**c**) succinate, (**d**) fumarate, (**e**) a-ketoglutarate, (**f**) malate, (**g**) lactate. Bacteria (wild type—empty columns, mutants—filled columns) were cultivated in semi-restrictive temperatures, established for each mutant, and the efficiency of plating was measured as CFU/mL. The *dna*(ts) mutations and metabolites are indicated above graphs. The experiments were repeated at least in triplicates (SD indicated).

Since suppression of growth defects of the temperature-sensitive replication mutations was observed in *pta*, *ackA*, *pgi*, *tktB*, and *gpmA* CCM mutants ([5]; see also Figure 1), we asked whether the excess of chosen metabolites (that might potentially accumulate in metabolically defective cells) can improve the growth of replication mutants. The semi-restrictive temperatures were chosen based on previous report [5] to achieve decreased efficiency of plating by 2–3 orders of magnitude. Various concentrations of metabolites (pyruvate, acetate, fumarate, succinate, malate, lactate, and α-ketoglutarate) were added to the solid medium, which was followed by plating of relevant strains. The metabolites’ concentrations were chosen by monitoring of bacterial growth in liquid cultures at permissive temperatures and selecting concentrations which did not impair bacterial viability. No significant differences in viability and culture growth rate were observed at 30 °C between wild-type and mutant bacteria, as well as between wild-type cells growing at different temperatures, at any tested concentration of metabolites. As expected, efficiency of plating of the tested mutants at semi-restrictive temperatures were about 2–3 orders of magnitude lower than that of the wild-type strain in the absence of respective metabolites (Figure 1).

However, we found that particular metabolites were able to suppress effects of replication mutations, whereby, the efficiency of suppression depended on metabolite concentration (Figure 1). To achieve complete suppression (i.e., when the efficiency of plating of the mutant strain was at the level of wild-type strain) of the *dnaA46* mutation, 10 mM pyruvate, 10 mM acetate, 2.5 mM fumarate, 15 mM malate, 15 mM lactate, or 5 mM α-ketoglutarate was necessary. Complete suppression of the *dnaB8* mutation required 5 mM α-ketoglutarate, or 250 mM succinate, while partial suppression occurred in the presence of 10 mM lactate or 20 mM malate. For the *dnaC* mutation, the presence of 12.5 mM acetate, 5 mM α-ketoglutarate, 15 mM lactate, 0.5 mM fumarate, or 250 mM succinate could suppress the growth defect. The effect of the *dnaG* mutation was suppressed only in the presence of 5 mM α-ketoglutarate, though partial suppression by pyruvate was also observed. Complete suppression of the *dnaE486* mutation occurred at 5 mM acetate or 5 mM α-ketoglutarate, while pyruvate, lactate, malate, and succinate caused partial suppression. Finally, only partial suppression of the *dnaN159* mutation could be observed in the presence of succinate. These results indicate that the addition of various metabolites may efficiently suppress temperature sensitivity of different mutants in replication genes, estimated as the ability to form colonies at temperatures which normally (without any treatment) caused a decrease in viability of mutants by 2–3 orders of magnitude. Therefore, we asked whether other mutant phenotypic properties of the tested strains could also be suppressed in the presence of an excess of different metabolites.

### 3.2. Effects of Metabolites on Filamentation of the Replication Mutants at Elevated Temperature

**Figure 2:**
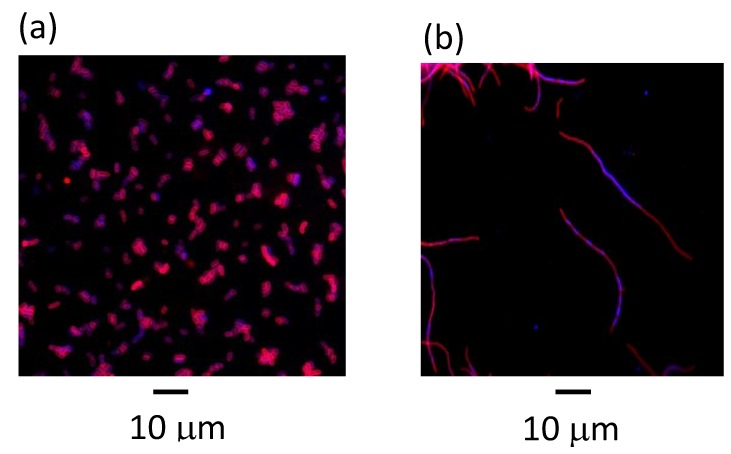

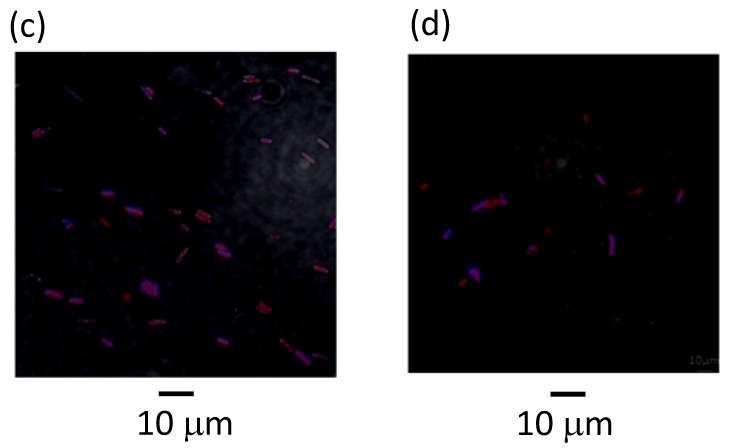
The microscopic analysis of the effect of CCM metabolites on bacterial cell morphology and nucleoid position. Wild type and *dnaA46* strains were incubated at 39 °C in the presence of the metabolite as indicated, prior to staining with DAPI and SynaptoRed. Bar indicates 10 mm. Panels represent: (**a**) MG1655 (wild-type), no metabolite added (**b**) *dnaA46*, no metabolite added (**c**) MG1655 (wild-type), lactate 20 mM (**d**) *dnaA46*, lactate 20 mM.

**Figure 3:**
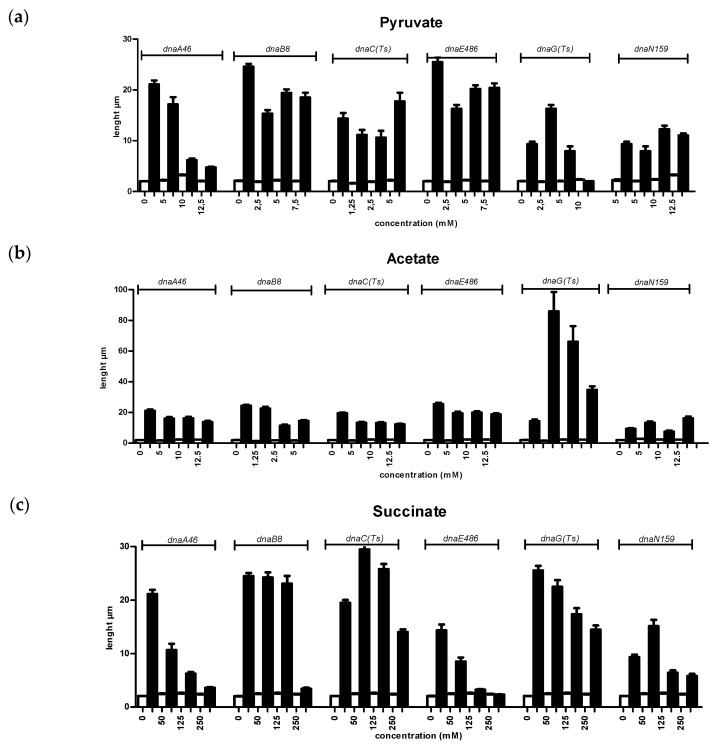

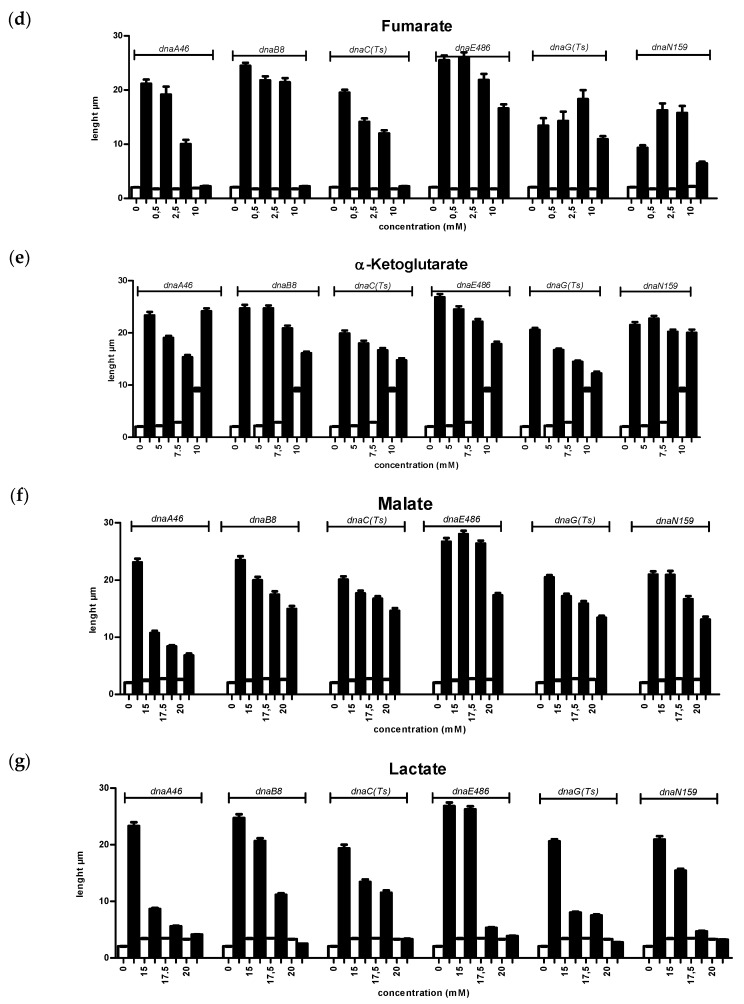
The effect of CCM metabolites on the cell length of replication mutants. Bacteria (wild type—empty columns, *dna*(ts) mutants—filled columns) were grown at semi-restrictive temperatures in the presence of following metabolites: (**a**) pyruvate, (**b**) acetate, (**c**) succinate, (**d**) fumarate, (**e**) a-ketoglutarate, (**f**) malate, (**g**) lactate. The cell length was measured for 100 bacteria per each strain. The *dna*(ts) mutations and metabolites are indicated above graphs. The experiments were repeated at least in triplicates (SD indicated).

Changes in environmental conditions lead to growth rate alterations. This effect could be connected to changes in the cell cycle, cell mass doubling, chromosome replication, and chromosome segregation in the bacterial cell. The replication deregulation may lead to severe defects in cell morphology (if cells do not divide correctly and make filaments) or in chromosomes’ segregation during cell division. Growth defect of replication mutants at the restrictive temperatures is reflected also in the impairment of cell division, leading to appearance of filamentous cells. Suppression of *E. coli* replication mutant phenotype in the presence of metabolic mutants included not only improvement of bacterial growth at restriction temperature [5], but also rescue of otherwise drastically elongated cell shape [17]. Therefore, we tested the effects of CCM metabolites on this parameter in the *dna*(ts) strains. Examples of the microscopic pictures of bacterial cells obtained in experiments with and without CCM metabolite supplementation of wild-type and *dna*(ts) cultures at 30 °C and the semi-restrictive temperature are presented in Figure 2. The results were quantitated by length measurements of 100 randomly chosen cells. At semi-restrictive temperatures, the length of the *dnaA46*, *dnaB8*, *dnaC*(ts), *dnaG*(ts), *dnaE486*, and *dnaN159* mutant cells varied from 10 to 30 μm, while it was 2–3 μm in the case of the wild-type strain (Figure 3). No significant differences between wild-type bacteria and mutants could be observed at 30 °C (in all cases, the length of cells was 2–3 μm). However, at semi-restrictive temperatures, effects of all the tested metabolites on mutant cell filamentation could be observed, although only some metabolites used at certain concentrations caused complete suppression of the filamentation phenotype. These conditions included: 12.5 mM pyruvate, 250 mM succinate, 10 mM fumarate, and 20 mM lactate for *dnaA46*; 250 mM succinate, 10 mM fumarate, and 20 mM lactate for *dnaB8*; 10 mM fumarate and 20 mM lactate for *dnaC*; 10 mM pyruvate and 20 mM lactate for *dnaG*; 250 mM succinate and 20 mM lactate for *dnaE486*; and 20 mM lactate for *dnaN159*. Other concentrations of metabolites caused partial suppression effects of various mutations in replication genes on cell shape (Figure 3). Moreover, changes in nucleoid localization, evident in mutants at semi-restrictive temperatures, were abolished by certain metabolites (Figure 2).

### 3.3. Kinetics of DNA Replication in the Presence of CCM Metabolites

To test whether the effects of CCM metabolites on *E. coli* replication mutants’ growth and filamentation at semi-restrictive temperatures are due to their direct and rapid action on DNA replication process, we aimed to estimate the efficiency of bacterial DNA synthesis in vivo.

**Figure 4:**
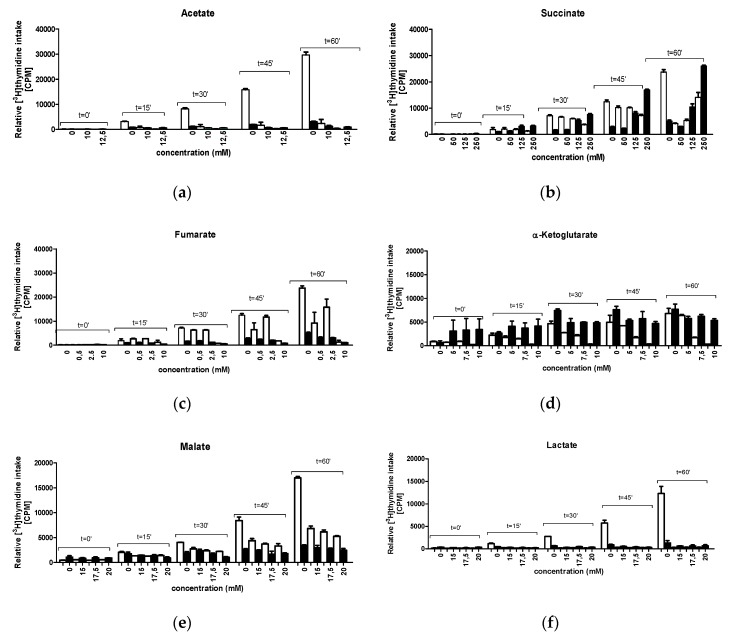
The kinetics of DNA replication in vivo in the presence of following CCM metabolites: (**a**) acetate, (**b**) succinate, (**c**) fumarate, (**d**) a-ketoglutarate, (**e**) malate, (**f**) lactate. The wild type (empty columns) and *dnaB8* (filled columns) bacteria were cultured at 41 °C. The DNA synthesis was assessed at time-points as indicated. The results are from at least three repetitions of experiments with SD indicated.

We measured the efficiency of incorporation of the labelled DNA synthesis precursor, [^3^H]thymidine, into the DNA. However, we found that the addition of vast majority of tested compounds did not improve the efficiency of DNA synthesis in mutants at semi-restrictive temperatures. Rather, inhibition of DNA replication by CCM metabolites was observed in both wild-type and mutant cells (Figure S1; note that for technical reasons, experiments were repeated three times separately for each metabolite. Therefore, efficiencies of ^3^H incorporation, expressed in CPM, may differ between experiments with different metabolites, and even values measured at time 0 may vary, which, however, did not affect the general picture and conclusions. Moreover, in some cases, particular metabolites caused strong inhibition of ^3^H incorporation, thus, effects of selected metabolites are demonstrated). The only exception was 250 mM succinate-mediated efficient improvement of DNA replication in the *dnaB8* mutant (Figure 4). Therefore, we conclude that in most cases, the observed effects of metabolites on mutants’ phenotypes were indirect.

## 4. Discussion

Direct links between the regulation of DNA replication and CCM have been reported in various organisms, from bacteria [3,5,17] to humans [7,8,9]. This implies that the modulation of DNA replication control by central carbon metabolism occurs commonly in both prokaryotes and eukaryotes [6]. Mediated by CCM, it appears that regulation of DNA replication might be of particular importance for all organisms, and defects in the CCM-replication regulation link may lead to serious problems in cell physiology, including eukaryotic cell transformation and formation of cancer cells [11]. On the other hand, molecular mechanisms of this link are largely unknown. Whether DNA replication regulation by CCM is based on protein-protein interactions or signals mediated by metabolites remains an unsolved question.

Only recently, some insights into understanding detailed mechanisms of the CCM-replication link in bacterial cells have been published. In *B. subtilis*, multiple links appear to connect CCM to DNA replication initiation and elongation [4]. It was suggested that changes in CCM may affect functions and/or recruitment of the DNA helicase, primase, and DNA polymerase at the replication origin, thus modulating the initiation stage, while the elongation process could be influenced due to changes in DNA polymerase operating at the replication fork. Although several CCM reactions have been suggested to influence DNA replication machinery, providing signals involved in the control of DNA synthesis at different stages [4], detailed signal transduction mechanisms remain unknown. In *E. coli*, it was demonstrated that temperature sensitivity of *dnaA46* mutant could be suppressed by defects in the acetate overflow pathway [18]. Such suppression is correlated with accumulation of pyruvate due to dysfunction of pyruvate dehydrogenase activity. In fact, double mutants, revealing thermosensitivity of DnaA initiator protein and lacking activity of pyruvate dehydrogenase, could grow at elevated temperature as efficiently, as wild-type strains [18].

Taking into consideration the above mentioned results, in this work, we tested the effects of various CCM metabolites (pyruvate, acetate, α-ketoglutarate, lactate, malate, fumarate, succinate) on temperature-sensitive phenotype of several *E. coli* mutants in replication genes including *dnaA*, *dnaB*, *dnaC*, *dnaG*, *dnaE*, and *dnaN*, coding for the replication initiator protein, DNA helicase, DnaA-DnaB linker, DNA primase, subunit of DNA polymerase III, and sliding clamp of DNA polymerase III, respectively. We found that various metabolites can suppress the growth defects of temperature-sensitive mutants at semi-restrictive temperatures. This suppression was observed as both an improvement in the efficiency of plating (Figure 1) and reduction of cell filamentation (Figure 2 and Figure 3). In this work, we not only confirmed previous observation that excess of pyruvate results in suppression of the *dnaA46* mutation [18], but also extended this phenomenon to other metabolites and other mutations. Regarding *dnaA46*, thermosensitivity of bacteria bearing this mutation could be suppressed by mutations in either *pta* or *ackA* genes, coding for phosphate acetyltransferase and acetate kinase, respectively (see Table 1). Dysfunctions of these genes may cause accumulation of acetate in cell, either indirectly or directly, respectively. Hence, suppression of effects of the *dnaA46* mutation by excess of not only pyruvate (as reported previously, [18]) but also acetate (Figure 1 and Figure 3) is compatible with previous observations [5], as well as with the hypothesis that the suppression in mediated by metabolites. Dysfunctions of genes coding for 2,3-bisphosphoglycerate-dependent phosphoglycerate mutase, glucose-6-phosphate isomerase, and transketolase 2, which were reported previously to suppress effects of temperature sensitivity of certain *dnaB, dnaE, dnaG,* and *dnaN* mutations in different combinations [5], lead to accumulation of various metabolites as these enzymes operate at key stages of CCM. Therefore, the data indicating suppression of growth and cellular morphology defects by different metabolites (Figure 1 and Figure 3), observed in corresponding *E. coli* replication mutants, are also compatible with previous results [5] and they can support the above presented hypothesis.

As discussed above, our data suggests that in *E. coli*, the CCM-replication link, is mediated by small metabolites rather than by direct protein-protein interactions. On the other hand, the excess of metabolites could not suppress defects in the direct DNA synthesis of the mutants, at least in short-term experiments (up to 60 min) (Figure 4). Therefore, the effects of metabolites on DNA replication appear to be indirect. The pattern of suppression effects also corroborates this conclusion. The mutants that were most responsive to metabolites included *dnaA46* and *dnaE486*, which harbor defects in the main proteins involved in DNA replication initiation and elongation, respectively, and are subject to extensive regulatory impacts. The least responsive mutant was *dnaN159*, bearing a change in the clamp of DNA polymerase III, which is an essential protein for DNA replication, but appears rather uninvolved in extensive control mechanisms. 

In summary, we demonstrated that various CCM metabolites suppress the effects of mutations in different replication genes of *E. coli*, providing evidence that the CCM-replication regulation link is mediated by small metabolites rather than direct protein-protein interactions. This corroborates the recent discovery that in *B. subtilis*, there are multiple links connecting CCM to DNA replication initiation and elongation [4,19]. Therefore, one may suggest that molecular mechanisms of CCM-dependent regulation of DNA replication are similar in *E. coli* and *B. subtilis*, making this regulation an important common feature of the control of cell physiology in bacteria. Since CCM-DNA-replication link has been also reported in human cells [7,8,9], we speculate that this regulatory mechanism might reflect a general biological adaptation, evolutionarily conserved from bacteria to humans.

## Figures and Tables

**Table 1:** *E. coli* replication and CCM genes and proteins involved in the replication-metabolism link.

Gene	Protein (Gene Product)	Protein Activity
*dnaA*	DnaA; replication initiator	Binding to the replication origin
*dnaB*	DnaB; DNA helicase	DNA unwinding in replication forks
*dnaC*	DnaC	Delivery of DnaB to the origin
*dnaE*	DnaE; a subunit of DNA polymerase III	DNA synthesis during replication
*dnaG*	DnaG; primase	Synthesis of primers during replication
*dnaN*	DnaN; b subunit of DNA polymerase III	Formation of the clamp conferring processivity during replication
*ackA*	Acetate kinase	Formation of acetyl phosphate from acetate and ATP
*gpmA*	2,3-bisphosphoglycerate-dependent phosphoglycerate mutase	Interconversion of 2-phosphoglycerate and 3-phosphoglycerate
*pgi*	Glucose-6-phosphate isomerase	Isomerization of *aldehydo*-D-glucose 6-phosphate to *keto*-D-fructose 6-phosphate
*pta*	Phosphate acetyltransferase	Interconversion of acetyl-CoA and acetyl phosphate
*tktB*	Transketolase 2	Production of xylulose-5-phosphate and ribose-5-phosphate from sedoheptulose-7-phosphate and glyceraldehyde-3-phosphate

**Table 2:** *E. coli* strains employed in this work.

Strain	Genotype	Reference
MG1655	F^-^ l^-^ *ilvG rfb-50 rph-1*	[12]
dnaA46	F^-^ l^-^ *ilvG rfb-50 rph-1 dnaA46 tna*::Tn*10*	[13]
dnaC(ts)	*leu thy rpsL dnaC*(ts)	[14]
dnaB8	F^-^ l^-^ *ilvG rfb-50 rph-1 dnaB8* cm^R^	[5,14]
dnaE486	F^-^ l^-^ *ilvG rfb-50 rph-1 dnaE486 zae502*::Tn*10*	[5]
dnaG(ts)	*leu thy rpsL dnaG*(ts)	[15]
dnaN159	F^-^ l^-^ *ilvG rfb-50 rph-1 dnaN159 zid501*::Tn*10*	[16]

## Supplementary Materials

The following are available online at https://www.mdpi.com/2073-4425/11/4/447/s1, Figure S1: The kinetics of DNA replication in vivo in the presence of CCM metabolites. The wild type (empty columns) and *dna*(ts) (as indicated, filled columns) bacteria were cultured at the semi-restrictive temperatures in the presence of indicated metabolites at various concentrations. DNA synthesis was assessed by measurement of [^3^H]thymidine incorporation at time-points as indicated. The results are from at least three repetitions of experiments with SD indicated.
