# Supplementary material for: The Role of Metabolites in the Link between DNA Replication and Central Carbon Metabolism in Escherichia coli
Source: Genes (Basel). 2020 Apr 19;11(4):447. doi: 10.3390/genes11040447 (PMC7231150; doi:10.3390/genes11040447)
Supplement: Supplementary file 1 [file genes-11-00447-s001.zip › CCM-dna-2020-(Genes)-Figure-S1-z.pdf]

Figure S1

The kinetics of DNA replication in vivo **in the presence of CCM metabolites**. The wild type (empty columns) and *dna*(ts) (as indicated, filled columns) bacteria were cultured at the semi-restrictive temperatures in the presence of indicated metabolites at various concentrations. DNA synthesis was assessed by measurement of [^{3}H]thymidine incorporation at time-points as indicated. The results are from at least three repetitions of experiments with SD indicated.

dnaA46

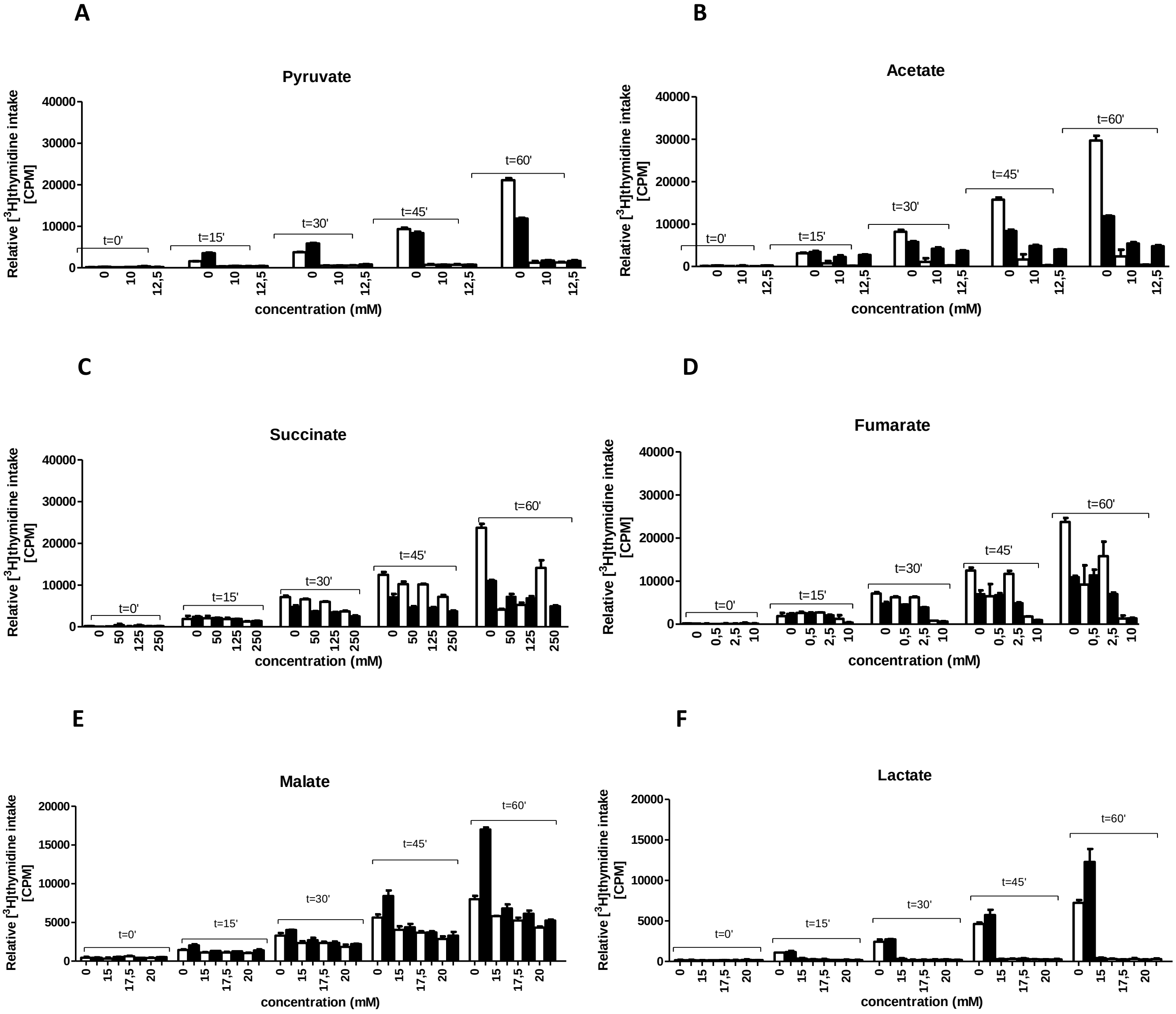

dnaC(ts)

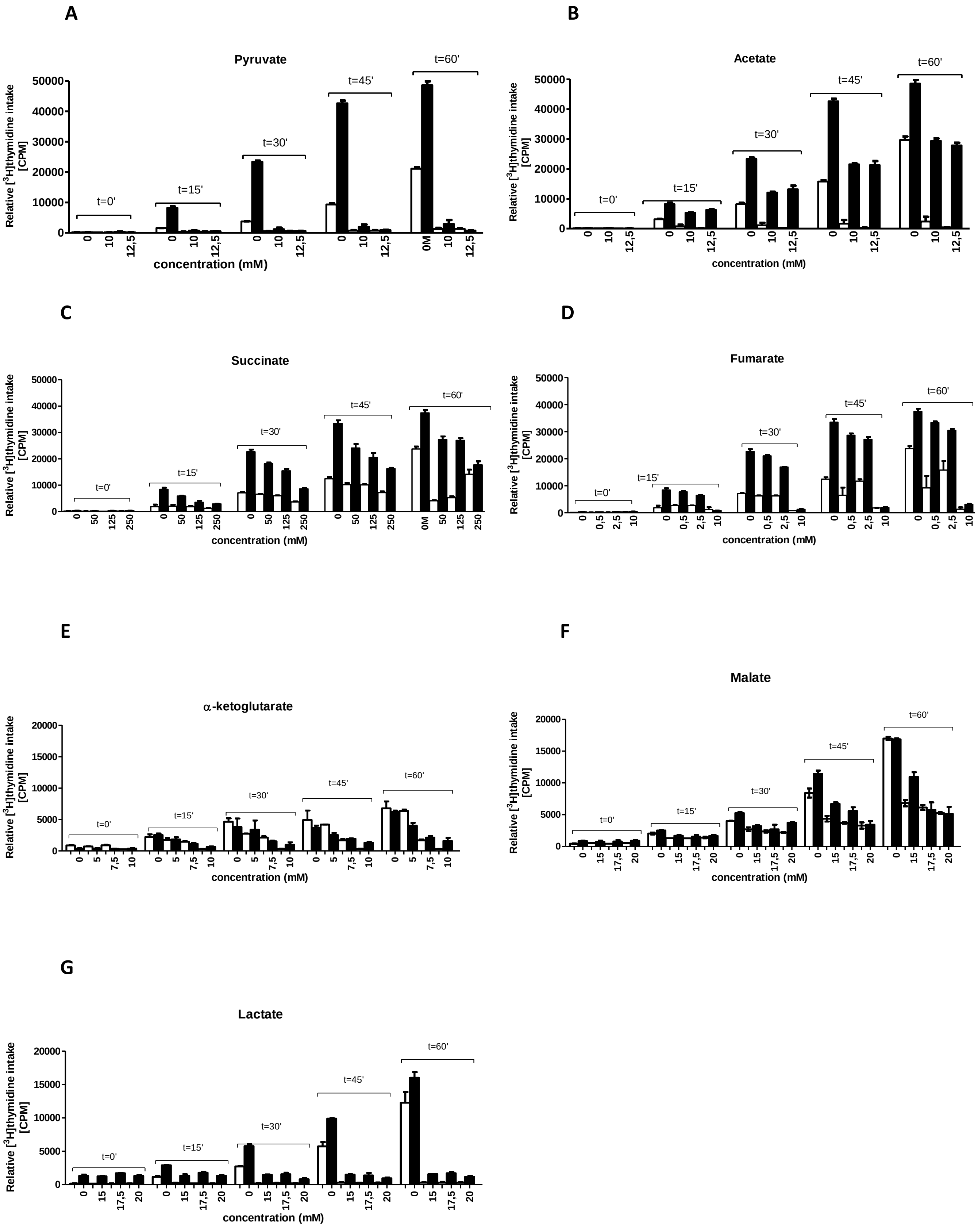

A
Pyruvate
B
Acetate
C
Succinate
D
Fumarate
E
α-ketoglutarate
F
Malate
G
Lactate
t=0'
t=15'
t=30'
t=45'
t=60'
Relative [3H]thymidine intake [CPM]
concentration (mM)

dnaE486

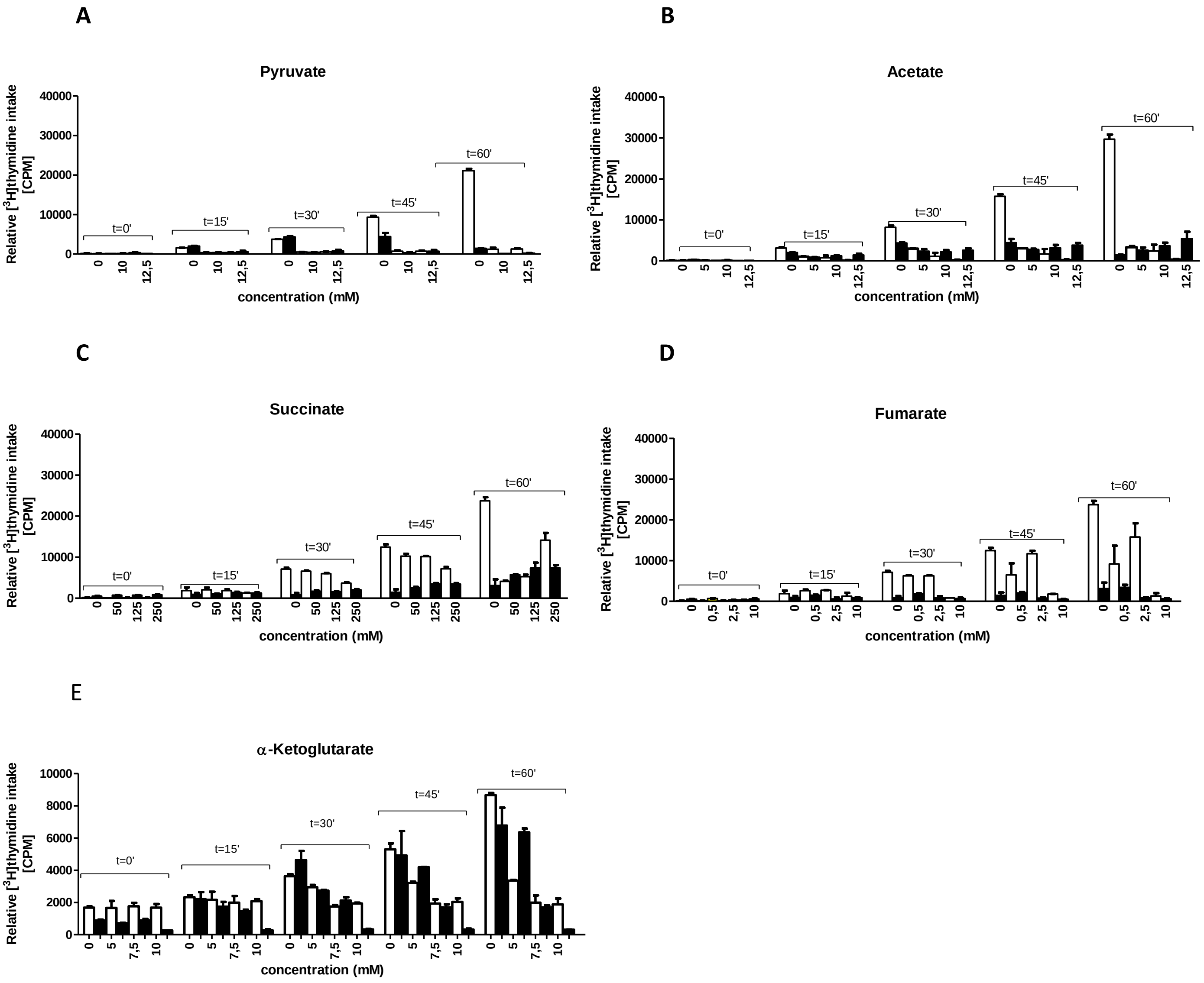
A
Pyruvate
Relative [3H]thymidine intake [CPM]
40000
30000
20000
10000
0
t=0'
t=15'
t=30'
t=45'
t=60'
0 10 12,5
concentration (mM)
B
Acetate
Relative [3H]thymidine intake [CPM]
40000
30000
20000
10000
0
t=0'
t=15'
t=30'
t=45'
t=60'
0 5 10 12,5
concentration (mM)
C
Succinate
Relative [3H]thymidine intake [CPM]
40000
30000
20000
10000
0
t=0'
t=15'
t=30'
t=45'
t=60'
0 50 125 250
concentration (mM)
D
Fumarate
Relative [3H]thymidine intake [CPM]
40000
30000
20000
10000
0
t=0'
t=15'
t=30'
t=45'
t=60'
0 0,5 2,5 10
concentration (mM)
E
α-Ketoglutarate
Relative [3H]thymidine intake [CPM]
10000
8000
6000
4000
2000
0
t=0'
t=15'
t=30'
t=45'
t=60'
0 5 7,5 10
concentration (mM)

dnaG(ts)

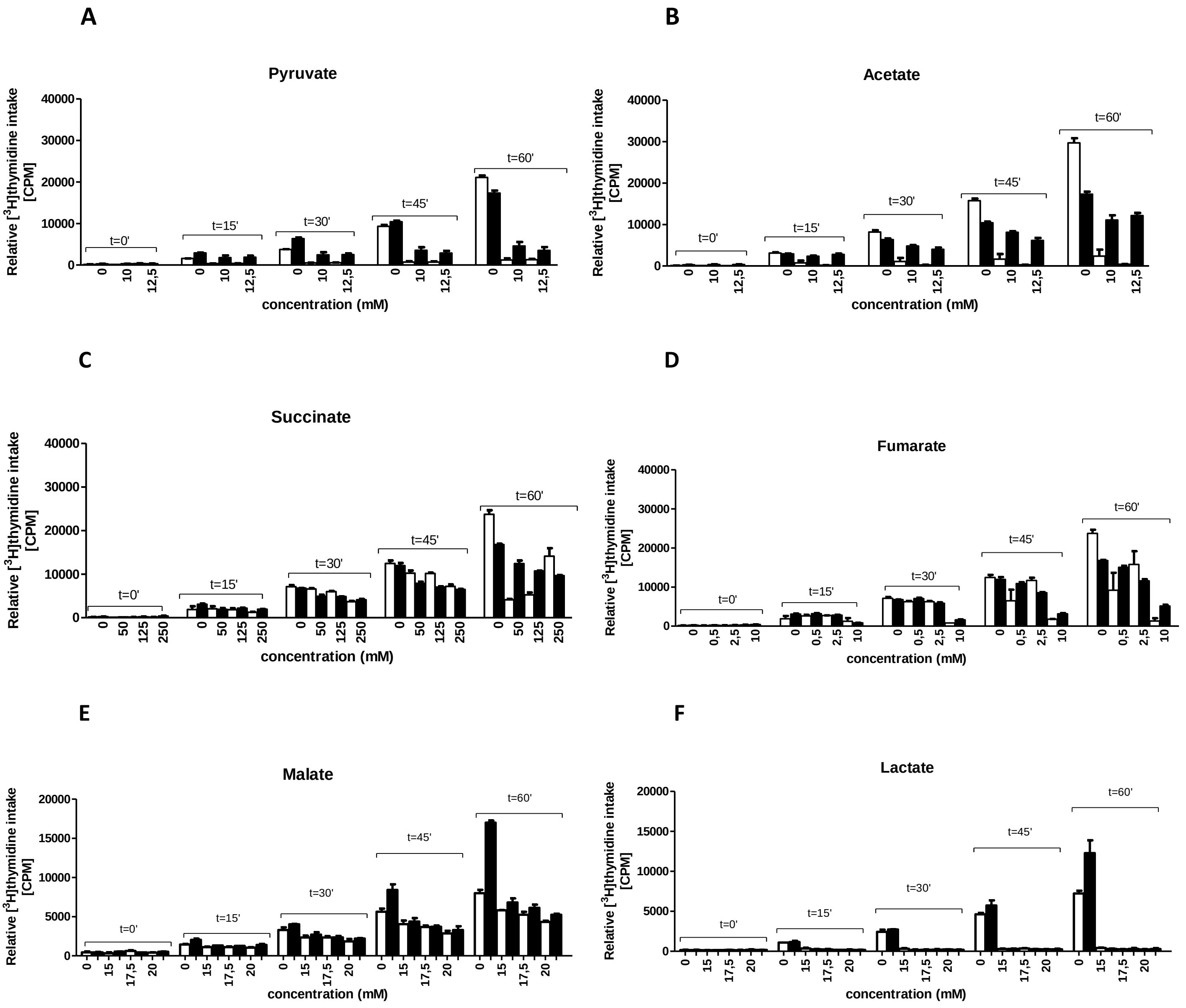
A
Pyruvate
B
Acetate
C
Succinate
D
Fumarate
E
Malate
F
Lactate
Relative [3H]thymidine intake [CPM]
concentration (mM)
t=0'
t=15'
t=30'
t=45'
t=60'

dnaN159

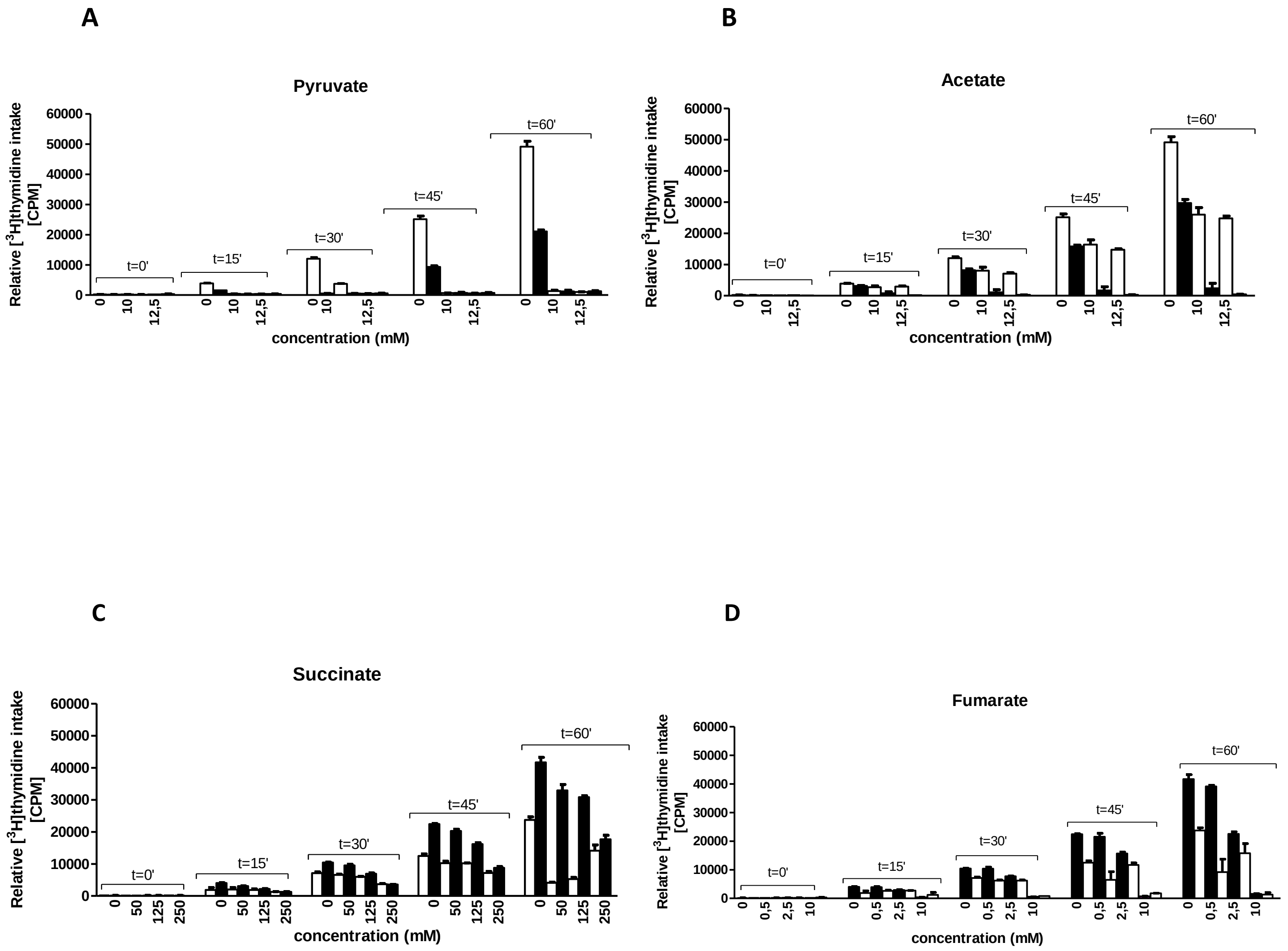
A
Pyruvate
Relative [3H]thymidine intake [CPM]
0
10000
20000
30000
40000
50000
60000
t=0'
t=15'
t=30'
t=45'
t=60'
0
10
12,5
concentration (mM)
B
Acetate
Relative [3H]thymidine intake [CPM]
t=0'
t=15'
t=30'
t=45'
t=60'
0
10
12,5
concentration (mM)
C
Succinate
Relative [3H]thymidine intake [CPM]
t=0'
t=15'
t=30'
t=45'
t=60'
0
50
125
250
concentration (mM)
D
Fumarate
Relative [3H]thymidine intake [CPM]
t=0'
t=15'
t=30'
t=45'
t=60'
0
0,5
2,5
10
concentration (mM)
